# Supplementary material for: Prevalence of Cryptococcal Antigenemia and associated factors among HIV/AIDS patients on second-line antiretroviral therapy at two hospitals in Western Oromia, Ethiopia
Source: PLoS One. 2019 Dec 5;14(12):e0225691. doi: 10.1371/journal.pone.0225691 (PMC6894769; doi:10.1371/journal.pone.0225691)
Supplement: S1 Supporting information — (DOCX) [file pone.0225691.s001.docx]

## Questionnaires used to collect data from HIV/AIDS patients

Wollega University

College of Medical and Health Sciences

Department of Medical Laboratory Sciences

Questionnaire for data collection on Cryptococcus infection among HIV-infected patients on second line ART Therapy at Western Oromia, Ethiopia

1. Name of the Hospital______________________
2. Participant Code _________________________
3. Data collector name_______________________
4. Contact persons: Nuguse G (0911016972)

NB: After asking whether the participant has not taken anti-fungal drugs within the past three months, please go to the questionnaires.

| **Part I: Socio-demographic Characteristics of the study participants** | | | |
| --- | --- | --- | --- |
| **S.N** | **Variables** | | **Response Category** |
| 301 | Age of the respondent in year__________________ | | |
| 302 | Sex of the respondent | - - 1. Female     2. Male | |
| 303 | Marital status | - - 1. Single     2. Married     3. Divorced     4. Widowed  1. Separated 2. Others (specify)_____ | |
| 304 | Educational status | - - 1. Illiterate     2. Read and writes     3. Primary school  1. Secondary school 2. Diploma and above | |
| 305 | Residence | - - - 1. Rural       2. Urban | |
| 306 | Average monthly income of the family in Ethiopian birr_______________? | | |
| 307 | Occupation | - - - - 1. Farmer         2. Merchant         3. Student         4. House wife  1. Employee 2. Daily labourer 3. Other(specify)________ | |

| **Part II: Clinical Sign and Symptoms, and associated risk factors related data** | | | |
| --- | --- | --- | --- |
| 308 | Date confirmed HIV+: ______/_______/_________(DD/MM/YYYY) | | |
| 309 | Time since HIV testing positive ( in year or month):____/_____/____ | | |
| 310 | ART initiated date of the patient: | - Date started First line ART_____/____/____ - Date started Second line ART ____/____/____ | |
| 311 | Months on ART: | - On 1st line ART_______________ - On 2nd line ART ______________ | |
| 312 | Clinical Adherence of patients during current visit | - - - 1. Poor       2. Good | |
| 313 | Distance of Health Facility from patients house | - - - - 1. <1 Km         2. 1-3 Km         3. >3 Km | |
| 314 | Did you ever have occupational exposure to blood? | - - - 1. Yes       2. No | |
| 315 | Did you ever have occupational exposure to soil? | Yes  No | |
| 316 | Do you have domestic animals (like hen)? | - - - 1. Yes       2. No | |
| 317 | If yes to Q. no. 316, the house of the household is: | 1. Not separated from domestic animals/hen 2. Separated from domestic animals/hen | |
| 318 | Did you ever have the following sign and symptoms?  You can select more than one response. | 1. Fever 2. Headache 3. Skin lesion 4. Cough 5. Neck stiffness or pain 6. Weight loss 7. Night sweat 8. Blurred vision | |
| **Part III: Laboratory Finding** | | | |
| 319 | *Cryptococcus neoformans* LFA test result of the patients | | 1. . Negative 2. . Positive |
| 320 | Baseline CD4 counts of the patient (from registration): _________________ | | |
| 321 | Latest/ Current CD4 counts of the patient: _______________________ | | |
| 322 | Number of HIV RNA Copies per ml or Viral Load test result:__________________ | | |

## Annex II: Consent Form

Topic: Prevalence of *Cryptococcal Antigenemia* among HIV-infected patients on second line ART at two Hospitals of Western Oromia, Ethiopia.

Principal Investigator: Nuguse Geda

Organization: Wollega University, College of Health Sciences

Sponsor: Self

Purpose of the Research Project:

The aim of this study is to determine prevalence of *Cryptococcal Antigenemia*and associated factors among HIV-infected patients on second line ART at the two Hospitals of Western Oromia, Ethiopia.

Procedure:

The study involved primarily interviewing sampled population living in West Shoa Zone and East Wollega Zone with Structured questionnaire, CrAg LFA & Viral load test had performedby takingblood samples from participants. Four trained hospital staff (two BSc nurses and two Medical Laboratory technologists) was participated for this data and sample collection purpose.

Benefits, Risk and /or Discomfort:

There is minimal risk (blood sample) associated and patients may benefit from this project if results suggest need for further investigation or follow up.

Incentives/Payments for Participating:

The participants haven’t provided any incentives or payment to take part in this project.

Confidentiality:

The personal information collected from the individual participants was kept confidential and stored in a file, without their names by assigned a code number to it.

Right to Refusal or Withdraw:

Participants have the full right to refuse participating and withdraw at any time in this research.

Person to contact:

This research project will be reviewed and approved by the ethical review committee of Wollega University. If you have any question, you can contact the following individuals at any time.

Nuguse Geda (WU MSc in Microbiology Student) Tel No – 0911016972,

Email address: nigusegeda72@gmail.com

Annex 2.1: Information to the Participant

Interview code no ________

Greeting and self-introduction and consent

Greeting: - Good morning/afternoon.

My name is Mr Nuguse Geda. We are conducting a scientific research on prevalence of *Cryptococcus infections* among HIV positive patients on 2^nd^ line ART and its associated risk factors that affect residents of Western Oromia region, Ethiopia. Therefore, I am happy to inform you that you are selected as one of the participants in this study. By participating in this research project you may feel some discomfort in wasting your time. However, your participation is definitely important in identifying the factors that are associated with prevalence of Cryptococcosis that will be helpful for your health, as you may not have symptom while having the disease. The interview may take 30-45 minutes and the information gathered will be used for writing a proposal for partial fulfilment of a specialty certificate in MSc in Microbiology at Wollega University.

Here, I want to assure you that any information obtained from you will remain confidential and even there is no need of writing your names or any personally identifiable information. There is no risk or direct benefit in participating in this research project. Your participation is determined only by you. If it is your willing; I will proceed to ask you some information. Finally, you are kindly requested to give your genuine response in the interview.

Certificate of Consent:

Do you wish to participate in the study? Yes/no

If the participant agrees to participate in the study, let him/her to sign consent and proceed with interview.

I have adequate information about the research and I have decided to participate in the study.

Signature -----------------------------------

If the participant says “No, I don’t want to participate in the study”, thank him/her and proceed to the next participant.

Name of interviewer_______________________

Date_____/_____/____

Annex 2.2: Informed Consent in Amharic:

**ለተሳታፊየሚሰጥየጥናትውልማስገንዘቢያ**

እኔ----------------------------------------------- የተባልኩየኤም.ፒ.ኤችየመመረቂያፅሁፌንለመስረትለሚየያስፈልገኝጥናትርዕስመመረጠንሳሳውቆትበታላቅደስታነው፡፡

ጥናቱየሚካሁደውበቃለነጠየቅመሰሰሪያእናጥቂትየደምናሙናበመውሰድሲሆንበእርሶላይምንምአይነትጉዳትአያደርስም፡፡ ከጥናቱመውጣትከፈለጉበማንኛውምስዓትአቋርጦመውጣትይችላሉ፡፡ ይህንበማድረጎውምንምአይነትተጽዕኖአይደርስቦትም፡፡ ከጥናቱየሚገኘውውጤትወደፊትለሚካሄዱሌሎችጥናቶችመነሻከመሆኑምባሻገርየተፈለገውጥናትበዞናችንእናበክልላችንምንእንደሚመስልያሰገነዝባል፡፡ የእርሶስምናሌሎችየእርሶንማንነትየሚያመላክቱነገሮችበጥናቱላይአይገቡም፡፡

የተሳታፊውፊርማ

አመሰግናለው!

ተሳታፊውበጥናቱለመሳተፍካልፈለገአመስግነውያስናብቷቸው፡፡

የጠያቂውስም----------------------------------------- ቀን------------------------------

Annex 2.3: Informed Consent in AfaanOromoo

I) OddeffanooQoratamaafkennamu

AniNiguseGedanjedhama.Karoorabarreeffamaeebbaairrattihirmaataaakkanaaftaatankabajaaninisingaafadha.Qorannoonkunkanadeemsifamuwaa’eedhukubafaangasiinamootajarmiin HIV dhiigaisaaniikeessattiargamurrattiilaalchiseyoota’u, hangarakkinadhibeen kun uumuu fi faca’inadhukkubni kun hawassalixaOromiyaakeessattiqabuadddaanbaasuu fi baruudha. Qorrannoonkundaqiiqaa 30 fudhachuudanda’a. Oodeffannoonqorrannookanarraaargamuhojiifuundurrattiadeemsifamuufgargaarsaguddaakenna.Qorrannookana keessaayeroobarbaaddanittiba’uukandandeessanyoota’u, kun immootajaajilaisiniikeennamuirrattidhiibbaatokkoilleehinqabu.

II) MallattooMirkaneessaa

Qo’annaa kana irratiqoodafudhachuufyoowaliigaltanbakkaarmaangadiiirratimallattookeessaniinmirkaneessaa.

Mallattooqoratamaa_________

Galatoomaa!

MaqaaQorataa--------------------------- Guyyaa------------------------------------

Yooqorannaa kana irrattiqoodafudachuuhinbarbaadneta’an,isaangalateeffadhaatiigaggeessaa.
