# Supplementary material for: Prevalence of Cryptococcal Antigenemia and associated factors among HIV/AIDS patients on second-line antiretroviral therapy at two hospitals in Western Oromia, Ethiopia
Source: PLoS One. 2019 Dec 5;14(12):e0225691. doi: 10.1371/journal.pone.0225691 (PMC6894769; doi:10.1371/journal.pone.0225691)
Supplement: Attachment — Submitted filename: Rebutal letter.docx [file pone.0225691.s002.docx]

**Dear editor and reviewer**

First of all, we would like to thank you for providing the reviewers’ comments which are very constructive and crucial. We have incorporated all the point by point below. The manuscript is formatted according to PLOSONE guidelines. We have cleaned the manuscript as well. We have highlighted “red” the changes made in the manuscript and we have a separate file for “manuscript with track changes” and a clean “Manuscript”. We have also attached a “rebuttal letter” which responds editor and reviewers’ comments and/suggestions point by point. We want the authorship order in the manuscript, not in the editorial manager, to be considered as final. Here below is a a “Rebutal letter” addressing reviewer comments point by point. Top of Form

**Response to the reviewer**

1. Authorship order is different on the manuscript than in the PLoS One system.

**Response**: We have communicated with the journal office for another paper and confirmed the authorship order in the manuscript is the final acceptable order. The journal has similar rules which we thought will be applicable for this manuscript, too. It was difficult for the corresponding author to put his name last in the editorial manager.

2. Abstract Results: would prefer you to specify the median age (with interquartile range). % married is of no value and is distracting. Would remove that.
   % living with HIV and/or on ART -consider changing units to years instead of months.

**Response**:  The age was described with median and IQR and we removed % marriage. And we changed unit of stay with HIV or ART in years instead of months.

3. Introduction: Line 59 -Define MLICs Line 61 -CrAg isn't antibodies, it's antigen. would remove the term anti-cryptococcal antibodies? replace with "antigen", same with line 63

**Response**: We defined MLICs (Middle and low income countries) and HICs (High income countries). We removed the term “anti-cryptoccocal antibodies” and changed the “antibody” miswording to “antigen”.

4. Methods:
   Line 90 -don't use contractions in formal writing. Same with line 132.

**Response**: We fixed the abovementioned issue.

5. Results: Line 162: don't capitalize "second line ART"
   Line 162 -saying 35% were in one age group is unhelpful. State the median age and interquartile range.

**Response**: We modified the phrase “Second Line ART” into “second line ART” and the median age was used than mean age.

6. Line 168 -change to years. Line 170 -is this an error? the baseline CD4 was 163 +/- 182? That doesn't make sense. Line 173 -the prevalence is 7.7%. is this among people with CD4<100? On line 170 it says mean CD4 was 434. Very confused about the population you're describing.

**Response**: We changed months to years in line 168. Line 170…it was error. Baseline CD4+ T cell count was 163 +/- 82, not 163 +/- 182. In line 173….the prevalence of CrAg test positivity was 7.7% from all study participants. But 50% prevalence was among participants whose current mean CD4+ T cell count <100 cells/µL. plus the phrase saying “….CD4 was 434….” is about the current mean CD4 T cell count. Thus our study population are those with current mean CD4 T cell count of 434, but their baseline (before starting treatment) was 163+/-82.

7. Line 221: Men tend to have more advanced HIV disease (ie. lower CD4 counts). Line 229: It's not clear from your results what the CD4 counts were of your patient population and those who were CrAg+

**Response**: Line 221…it doesn’t necessarily mean men has advanced disease than women rather men usually have outdoor activities in Ethiopia which exposes them for Cryptococcosis infection which in turn resulted higher prevalence of CrAg test positivity among men. Line 229……We included the proportion of CrAg positive with respect to CD+ T cell count in results section.

8. Line 257: higher than what?Bottom of Form

**Response**: Line 260….higher. it is corrected into “….was significantly ‘high’ in the study area”.

9. In your figure, label your Y axis. Don't use decimal places unless it's useful. And put percentages behind your numbers.

**Response**: The figure was modified accordingly Close.OKCancel

ErrorClose

OK

 Close

OK
